# Inequalities in health care utilization among migrants and non-migrants in Germany: a systematic review

**DOI:** 10.1186/s12939-018-0876-z

**Published:** 2018-11-01

**Authors:** Jens Klein, Olaf von dem Knesebeck

**Affiliations:** Institute of Medical Sociology, University Medical Center Hamburg-Eppendorf, Martinistr. 52, 20246 Hamburg, Germany

**Keywords:** Migration, Ethnicity, Inequality, Utilization, Access, Health care, Germany, Systematic review

## Abstract

**Background:**

Despite the growing number of people with migrant background in Germany, a systematic review about their utilization of health care and differences to the non-migrant population is lacking. By covering various sectors of health care and migrant populations, the review aimed at giving a general overview and identifying special areas of potential intervention.

**Methods:**

A systematic review was conducted in PubMed database including records that were published until 1st of June 2017. Further criteria for eligibility were a publication in a peer-reviewed journal written in English or German language. The studies have to report quantitative and original data of a population residing in Germany. The appropriateness of the studies was judged by both authors. Studies were excluded if native controls were not originated from the same sample. Moreover, indicators of health care utilization have to assess individual behaviour like consultation or participation rates. 63 studies met the inclusion criteria for a qualitative synthesis of the findings.

**Results:**

The overall findings indicate a lower utilization among migrants, although the results vary in terms of health care sector, indicator of health care utilization and migrant population. For specialist care, medication use, therapist consultations and counselling, rehabilitation as well as disease prevention (early cancer detection, prevention programs for children and oral health check-ups) a lower utilization among people with migrant background was found. The lower usage was particularly shown for migrants of the 1st generation, people with two-sided migrant background, children/adolescents and women. Due to the methodological heterogeneity a meta-analysis was not feasible. As most of the studies were cross-sectional, no causal interpretations could be drawn.

**Conclusions:**

The inequalities in utilization could not substantially be explained by differences in the socioeconomic status. Other reasons of lower utilization could be due to differences in need, preferences, information, language and formal access barriers (e.g. charges, waiting times, travel distances or lost wages). Different migrant-specific and migrant-sensitive strategies are relevant to address the problem for certain health care sectors and migrant populations.

**Trial registration:**

The review protocol was registered on PROSPERO (CRD42014015162).

**Electronic supplementary material:**

The online version of this article (10.1186/s12939-018-0876-z) contains supplementary material, which is available to authorized users.

## Introduction

Health and health care among migrants are relevant and controversial issues in Europe since many years [1, 2]. The number of people with migrant status living in Europe is growing rapidly. In Germany, more than 20% of the total population of about 82.4 million people have a migrant background, resulting in about 18.6 million individuals (based on a definition of migrant background to people with foreign nationality, foreign country of birth, or if at least one parent is foreigner or was born abroad) [3]. In this context, health care access and utilization of migrants have become an essential subject [4, 5]. Ethnicity is an important determinant of health care utilization included in the well-established Behavioral Model of Health Services Use by Ronald Andersen [6–8]. His conceptual framework distinguishes between predisposing, enabling and need factors of health care use. Ethnicity is a contextual (ethnic and racial composition, spatial segregation) and an individual (nationality, country of origin) predisposing characteristic when analysing disparities in health care utilization [6]. Migrant populations may have different health care needs, preferences and expectations or face barriers to their use of health services. Previous studies and reviews about inequalities in health care utilization among migrants in Germany or Europe were restricted to particular somatic or mental health outcomes, health care sectors, specific migrant populations, were not systematic or did not include native controls to analyse disparities [9–15]. The aim of this review is to assess inequalities in health care utilization among migrants and natives in Germany. Three research questions are of main interest: (1) Are there inequalities in health care utilization between people with and without migrant background in Germany? (2) Are there differences regarding health care sector or indicator of health care utilization? (3) Are there differences in terms of particular migrant populations and type of migrant background?

## Methods

A systematic review was conducted based on the PRISMA guidelines [16]. The review protocol was registered on PROSPERO (CRD42014015162). The PubMed database was searched including all publications until 1st of June 2017 (for more information about the search strategy see Additional file 1). Furthermore, the search was completed by scanning the references of identified publications and an additional hand search. Inclusion criteria were as follows: eligible articles have to be published or in-press in a peer-reviewed journal and written in English or German language. The studies have to report quantitative and original data of a population residing in Germany, reviews or commentaries were excluded. The indicators of health care utilization have to assess individual behaviour like consultation or participation rates (i.e. studies using biomarker, health outcomes or disease stage at presentation as surrogate for utilization were excluded). Inequality between migrants and natives should be analysed by originating native controls from the same sample. Studies that compare results with normative data or other surveys to analyse inequalities were not included. Moreover, studies that only adjust for the migrant background as a confounder without explicitly reporting associations with health care utilization were excluded. Titles and abstracts of studies were screened by the two authors for eligibility. After the exclusion of studies that did not provide quantitative data on the relation between migrant status and health care utilization, full texts were retrieved. Disagreements on the exclusion of studies were discussed by the two reviewers until a consensus was found. In some cases, the investigators were contacted for further questions. Information extracted from the selected studies includes: first author and publication date, net sample size, area, year of data collection, operationalization of migrant background, indicator(s) of health care utilization, adjustments in the full model (if applicable), statistical measure (i.e., percentage, mean, odds ratio, relative risk etc.) and findings. In terms of adjustment, gender, age and socioeconomic status (SES) were considered as covariates in most studies. Results were specified when 1st and 2nd generation migrants or one- or two-sided migrant background were distinguished in the studies. Moreover, the qualitative assessment of the trend of inequality in the different health care sectors were summarised in a table. A meta-analysis including pooled estimates as well as an assessment of study quality and risk of bias was not conducted due to the number of studies and their heterogeneity concerning health care sectors (e.g. diverse outpatient care, inpatient care, rehabilitation and prevention programs) and methodological approaches (e.g. sample characteristics, outcome measurements, statistical analyses and reported effect estimates).

## Results

**Fig. 1:**
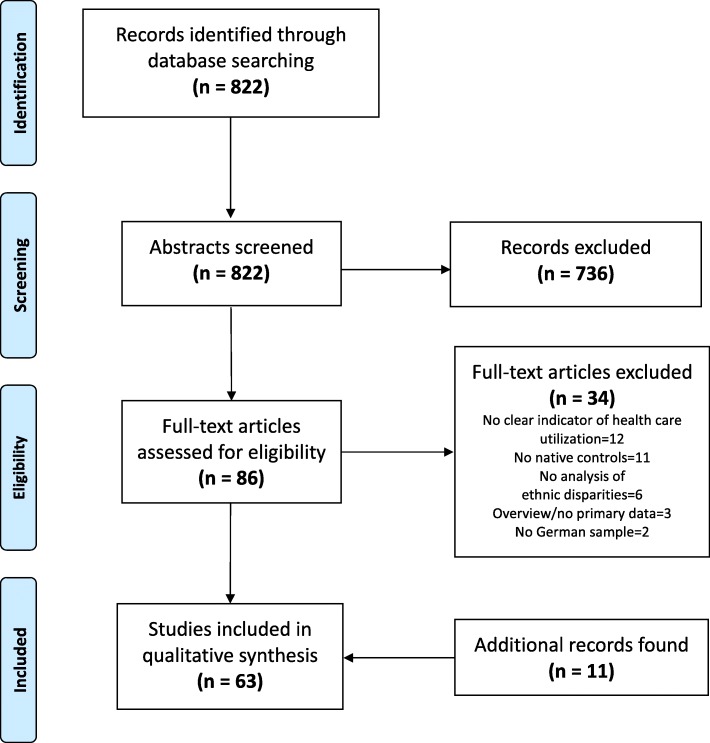
PRISMA flow diagram

The initial search in PubMed Database generated 822 records. After screening of titles and abstracts 86 publications remain for a full-text review. Thereof, 34 articles did not meet the inclusion criteria. Main reasons for exclusion were an ineligible indicator of health care utilization or the absence of native controls within the sample. Additionally, 11 records were found by screening the reference lists of all extracted articles and by hand search resulting in 63 studies finally included in the review. Detailed information is provided in the PRISMA flow diagram in Fig. 1.

The publications were categorised according to the following groups which are summarised in the Additional files 2, 3 and 4 including a more detailed presentation of measurements, statistical methods and findings:

- utilization of outpatient care (subdivided into unspecific physician consultation, general practitioner (GP)/paediatrician), specialist care, inpatient care, emergency care and rehabilitation (Additional file 2),
- utilization of therapists, counselling services and medication use (incl. Complementary and alternative medicine (CAM)) (Additional file 3), and
- utilization of disease prevention provided by a physician (i.e. early cancer detection, early detection program for children, vaccination, general health check-up, oral health check-up) (Additional file 4). Utilization of health promotion programs (e.g. behavioural prevention programs) was not included into the review.

### Outpatient care (physicians), inpatient care, emergency care and rehabilitation

Five publications examined associations regarding unspecific outpatient care (i.e. no distinction between GP/paediatrician and specialist) [17–21]. Overall, a slightly higher utilization among people with migrant background (PMB) was reported. The utilization of GPs and paediatricians was assessed by five studies showing small differences between PMB and the non-migrant population (NMP) [22–26]. The largest number of studies (*n* = 14) was found for specialist consultations [22, 23, 25–36]. The majority of the results showed lower utilization for PMB, especially among 1st generation migrants. For instance, one study showed a lower probability of utilization especially among 1st generation migrants (odds ratio (OR): 0.58; confidence interval (CI): 0.40–0.83) [23]. Inpatient care was assessed by the number of hospitalizations and elective surgeries (mostly caesarean section) [17, 18, 23, 25, 26, 29, 30, 37–39]. The 10 studies revealed an inconsistent pattern in terms of ethnical disparities. The small number of studies (*n* = 3) that examined emergency care utilization do not enable a definitive conclusion. One current publication showed higher utilization [40] among PMB while two others found no differences [26, 41]. Also, the four studies regarding the utilization of rehabilitation (out- and inpatient) provide insufficient evidence [26, 42–44], albeit the trend suggests a lower participation among PMB. A table with all details of the selected studies is provided in Additonal file 2.

### Therapists, counselling services and medication

The publications that investigated inequalities in using therapists and counselling services (*n* = 8) mostly reported lower utilization among PMB. This holds true for physical (adult 1st generation migrants, children and adolescents) [45, 46] and occupational therapy (children and adolescents) [47], psychosocial care/counselling [26, 28, 48], but not for the two studies analysing utilization of psychotherapists and psycho-oncologists [22, 49]. In terms of physical therapy, a recent multi-level analysis found a lower probability of utilization among 1st generation migrants (OR: 0.67; CI: 0.51–0.89) [45]. Medication use (especially self-medication/over-the-counter drugs) [50–52] and utilization of CAM [24, 26, 53–56] (13 studies) was more prevalent among NMP (adults as well as children and adolescents). There is no clear pattern in terms of prescribed drugs and off-label medicine (see Additional file 3 for more details) [51, 57–59].

### Disease prevention

Compared to the other health care sectors, there are more studies on migrant-specific utilization of disease prevention programs (in sum *n* = 34). In case of early cancer detection, five out of seven studies indicated lower participation for PMB [17, 26, 60–64]. Particularly, this holds true for migrants of the 1st generation or with a two-sided migrant background. Similarly, the participation in preventive health care programs for children aged zero to 5 years (named “u-examinations” in the German health care system) is consistently less prevalent among PMB [25, 65–68]. A different pattern was shown in terms of vaccination uptake. Subject to the type of vaccination, inequalities substantially vary, so a clear trend cannot be identified [41, 61, 66, 67, 69–77]. Only two studies examined ethnic inequalities in the utilization of the general health check-up that is offered to every person aged ≥35 years [26, 61]. PMB participated less often. Finally, consistent inequalities were shown when participation in oral health check-ups was investigated, with PMB showing lower rates in all age groups (see Additional file 4 for more details) [24, 26, 27, 58, 61, 78, 79]. For example, a recent publication among adults showed a significantly lower probability of utilization of oral health check-ups even in the fully adjusted model (OR: 0.71; CI: 0.65–0.77) [78].

A summary of the findings including number of studies, trend of inequality and description of trend for all health care sectors is provided in Table 1.

**Table 1:** Summary of findings

Sector of health care	No. of studies [references]	Inequality (trend)	Description of trend
Outpatient care (unspecific)	5 [17–21]	+	▪ Higher probability of utilization especially among migrant children and adolescents.
Outpatient care (general practitioner or paediatrician)	5 [22–26]	O	▪ No striking differences, but lower probability of utilization among migrant children and adolescents. ▪ Higher utilization among 1st generation migrants.
Outpatient care (specialists)	14 [22, 23, 25–36]	–	▪ Lower frequency and probability of utilization especially among 1st generation migrants. ▪ Inequality varies with the type of specialist.
Inpatient care	10 [17, 18, 23, 25, 26, 29, 30, 37–39]	O	▪ Inconsistent results, differences in terms of type of disease.
Emergency care	3 [26, 40, 41]	O	▪ Inconsistent results: Higher probability of utilization among migrant adults, but no difference among children and adolescents.
Rehabilitation	4 [26, 42–44]	–	▪ Results tend to lower usage among migrants. ▪ Two multivariate analyses indicate lower probability of utilization among people with migrant background.
Therapists and counselling services	8 [22, 26, 28, 45–49]	–	▪ Lower probability of utilization among 1st generation migrants (physical therapy) and migrant children of lower age groups (physical and occupational therapy). ▪ In terms of (psychosocial) counselling slightly higher frequency of uptake among natives, but no differences in the probability for cancer survivors.
Medication and complementary and alternative medicine (CAM)	13 [24, 26, 50–59]	–	▪ Lower frequency and probability of utilization among people with migrant background, especially for CAM and in case of self-medication (e.g. over-the-counter drugs). ▪ Unclear pattern in terms of prescribed drugs, but trend to higher use among migrants.
Early detection (cancer)	7 [17, 26, 60–64]	–	▪ Lower frequency and probability of participation especially among migrant women and migrants of the 1st generation or with two-sided background (any cancer sites).
Early detection (children)	5 [25, 65–68]	–	▪ Consistently lower frequency and probability of participation in the preventive health care program for children with migrant background.
Vaccination	13 [41, 61, 66, 67, 69–77]	O	▪ Inequality varies with the type of vaccination. ▪ In some cases, lower frequency and probability of utilization notably among 1st generation migrants, in other cases, lower uptake among natives.
General health check-up	2 [26, 61]	–	▪ Lower frequency of utilization among migrants, but small number of studies.
Oral health check-up	7 [24, 26, 27, 58, 61, 78, 79]	–	▪ Lower frequency and probability of utilization in all age groups of migrants.

+ = Higher frequency and/or probability of utilization among people with migration background

– = Lower frequency and/or probability of utilization among people with migration background

O = No consistent pattern/no difference

## Discussion

There are inequalities in health care utilization between migrants and natives in Germany. These disparities vary in terms of health care sector, indicator of health care utilization and migrant population. A lower utilization among PMB was particularly shown for specialist care, medication use, therapist consultations and counselling as well as disease prevention (especially early cancer detection, prevention programs for children and oral health check-ups). No disparities or inconsistent patterns were shown for GP/paediatrician visits, inpatient care and vaccinations. A low number of studies regarding emergency and general health check-up does not allow a clear statement. Usage of rehabilitation tends to be more prevalent among NMP. Furthermore, migrants of the 1st generation, people with two-sided migrant background, children and adolescents as well as women were identified as migrant groups with a particularly low utilization. Studies from other European countries are in line with some presented results. Two systematic reviews showed higher utilization of emergency departments by migrants [10, 11] while one indicated inconsistent results [12]. Furthermore, specialist visits and screening uptake were reduced among PMB in different countries [11, 12]. This also holds true for the utilization of mental health care [13].

Moreover, it is an important topic if the SES (income, education, occupational status) contributes to potential inequalities between migrants and non-migrants [80, 81]. Notably, the pattern of migrant-specific inequalities in health care utilization is quite similar to disparities in utilization regarding SES [82]. Some of the extracted studies aimed at analysing the contribution of SES concerning ethnic disparities in health care utilization [42, 62, 64]. Even after stepwise introduction of SES variables into the model, no or only a low reduction of the association between migrant background and health care utilization was shown for different health care sectors. A number of studies introduce various covariates (including SES variables) at once into the analysis, and both the estimates of migrant background and SES remain significant (data not shown in detail) [e.g. 40, 45, 51, 53, 78]. In other cases, the association with migrant background is significant while the association with SES is not [19, 72, 20]. These results suggest that in many cases, migrant background/ethnicity is a determinant of health care use, independent from SES, and associations between migrant status and health care utilization cannot substantially be explained by SES.

### Reasons for differences in health care utilization

Differences in health care utilization between migrants and non-migrants can imply acceptable or unacceptable inequalities [83]. The former include differences in expectations and preferences (e.g. individual/cultural preferences or health beliefs), the latter mean differences in information (e.g. about service availability), language/communication or (formal) access barriers (e.g. charges, waiting times, travel distances or lost wages). For the majority of the extracted studies, it is not possible to figure out the reasons for non-utilization of health care. In a German survey, language barriers, lack of information about the health care system and the assumption that cultural peculiarities would not be understood by the expert staff were indicated as the main reasons for a non-utilization (any health care sector) [84]. Another study including patients in a paediatric setting and health care providers found out that a major difficulty was the lack of mutual understanding, often associated with language barriers and difficulties in managing cultural diversity [85]. In mental health care, translation problems and misunderstandings based on divergent explanations in terms of the causes, course, and adequate treatment of different disorders were found to play an important role [86]. Furthermore, lower health literacy (i.e. knowledge, motivation and competences to access, understand, appraise, and apply health information) for migrants living in Germany than for German natives were shown for different age groups [87]. A lower utilization of, for instance, over-the-counter drugs could be due not only to lower health literacy but also to limited financial resources. Another barrier comprises factors of discrimination. US-American research examined that a higher prevalence of discrimination among ethnic minorities may contribute to their underutilization of health care services [88]. Finally, differences in health care needs could also be a reason for disparities in health care utilization [6, 83]. There is still a lack of evidence and unclear patterns about health care needs of migrants. Often, they are initially healthier compared with NMP (healthy migrant effect) while some data suggest that they tend to be more vulnerable to certain diseases [2, 89].

### Strengths and limitations

Generally, most of the studies were cross-sectional and had a descriptive and explorative approach. So, no causal conclusion can be drawn and most of the inequalities could not be explained by statistical data. Studies differ in the assessment and definition of migration status/background (e.g. country of birth, nationality, language spoken at home) including information about important subgroups (e.g. 1st/2nd generation, one- or two-sided background) and in recruitment strategies (register-based, community-orientated) [90, 91]. Therefore, comparability of the data is somewhat limited. Furthermore, some articles did not focus on migrants although results regarding migrant background as covariate was shown in the tables. Thus, the identification of eligible studies is hampered to a certain degree. The reference screening of the identified publications and the additional hand search was aimed to minimize that potential limitation. As mentioned in the Methods section, a meta-analysis including a risk of bias assessment and a pooled estimate could not be conducted due to the methodological heterogeneity of the selected studies. The qualitative synthesis of inequality was not based on a specific criterion. Moreover, the aforementioned adjustment of need (e.g. level of ill-health, capacity to benefit from health care or the expenditure a person ought to have) was not (or insufficiently) realized in the majority of the studies [83]. Often, the subjective health status was used as a proxy for need and this measure is amongst others known to be biased by ethnicity [92]. Finally, due to a comparatively small number of studies, evidence is not sufficient for conclusions in terms of some health care sectors (emergency care, rehabilitation, general health check-up).

To our knowledge, this is the first comprehensive systematic review that worked out migrant-specific inequalities in health care utilization for numerous health care sectors and migrant populations in Germany. The detailed and sensitive search strategy was aimed to identify all relevant publications and to provide evidence of ethnic disparities in German health care. However, it is possible that additional studies could not been identified, but it is assumed that additional results would not have altered the overall findings. Nevertheless, a publication bias could have limited the results as studies resulting in weak or no associations at all tend to be published less often.

### Implications and interventions

Generally, two different strategies are discussed to address migrant populations. ‘Exclusive’ migrant-specific strategies imply services and interventions specifically addressed at this group [93]. It is argued that differences in e.g. biology, life course, language, culture between migrants and natives require specific health and preventive interventions. Potential stigmata and the heterogeneity of migrant groups are named to be disadvantageous for this strategy. The ‘inclusive’ migrant-sensitive approach aims at adapting the existing routine health and preventive services to the specific requirements. Thus, the social determinants of health which affect all members of a society underlie ethnic disparities, whilst cultural differences would be overestimated [94]. There is strong evidence that social inequalities in health, health care access and utilization are a major topic for health policy in various countries [95, 96]. For the case of Germany, this review shows that patterns of ethnic inequalities in health care utilization are quite similar to inequalities according to SES (i.e. income-related, educational, occupational), but the ethnic disparities could not substantially be explained by the SES of the patients. There are controversial discussions if differences in SES are the essential explanation for migrant-specific disparities in health and health care [80, 81]. Indeed, the assumption that migration is an independent social determinant of health is becoming more and more popular [80, 97]. The complexity of social inequalities among ethnic minority groups cannot be fully captured by simple measures of SES. Specifics among migrants are a non-comparability of SES markers across ethnic groups, the importance of life course inequalities and other risk factors like racism and geographical segregation that may affect health. Finally, a mixture of health care programs that include (migrant-sensitive) and specifically address (migrant-specific) PMB is needed [98].

Beside the discussion of fundamental strategies, the aforementioned different aspects of non- or under-utilization implies different needs for action in practice. The German concept of “intercultural opening” implies reduction of communication barriers by using interpreters, mediating between divergent explanations, avoiding cultural stereotyping and supporting an open-minded, reflective professional approach [86, 99]. Language barriers are often mentioned to be a major problem for migrants when accessing and utilizing health care. Professional face-to-face interpretation, professional interpretation by telephone, informal face-to-face interpretation, bilingual professionals or cultural mediators (health workers) are methods for tackling language barriers [2]. There are attempts to overcome these barriers (e.g. in a German hospital setting), but the current resources seem not to be appropriate and sufficient [100]. A development of quality standards and the provision of financial resources are recommendations for a significant improvement. Moreover, the strengthening of migrants’ health literacy and knowledge of available care programs by native speaking counselling services, and developing culturally sensitive patient information material aim at improving health care access and utilization of PMB in Germany [101, 102]. The different ways migrants perceive health problems or face administrative requirements has to be of interest, too [2]. For the utilization of prevention or emergency care, tailored education measures on the general functioning of the German health systems should be provided for an improved patient empowerment [40, 98]. For instance, this could be included in integration classes that are offered to people migrating to Germany. Nevertheless, attention should be paid on established formal access barriers for socially deprived people like waiting times, co-payments, travel distances or lost wages [103]. A particular topic for research and intervention programs are the special needs and restricted entitlements for the growing number of refugees, asylum-seekers and undocumented migrants in the recent years [4, 104]. Studies specifically addressing these populations were included into the initial search, but were subsequently excluded due to unmet inclusion criteria [105, 106]. Their insecure residence permit status implies some more formal and informal barriers to access and utilize health care [4]. Experiences of war, devastation and escape leads to particular needs in the fields of psychosocial and mental health, chronic diseases and the provision of care to children [104, 107]. Despite the increased number of publications, a lack of valid data avoids evidence-based decisions.

## Conclusions

This comprehensive overview covers numerous health care sectors and migrant populations in association with inequalities of health care utilization in Germany. Overall, people with migrant background indicate a lower utilization of health care, but inequalities vary with health care sector and subpopulation of migrants. The SES cannot substantially explain migrant-specific inequalities. Hence, the enlisted aspects and complexity of migration, health care and their interaction need further efforts in research and practice. With respect to different areas of health care and vulnerable groups, various migrant-specific and migrant-sensitive interventions are required. The current available data provides some references for action.

## Additional files

Additional file 1: Search strategy. (PDF 168 kb)

Additional file 2: Overview of the characteristics of included studies (utilization of outpatient care (physicians), inpatient care, emergency care and rehabilitation). (DOCX 31 kb)

Additional file 3: Overview of the characteristics of included studies (utilization of therapists and counselling services, complementary and alternative medicine (CAM) and medication use). (DOCX 20 kb)

Additional file 4: Overview of the characteristics of included studies (utilization of disease prevention). (DOCX 28 kb)
