# Supplementary material for: Inequalities in health care utilization among migrants and non-migrants in Germany: a systematic review
Source: Int J Equity Health. 2018 Nov 1;17:160. doi: 10.1186/s12939-018-0876-z (PMC6211605; doi:10.1186/s12939-018-0876-z)
Supplement: Supplementary file 1 — Search strategy. (PDF 168 kb) [file 12939_2018_876_MOESM1_ESM.pdf]

Additional file 1: Search strategy

Database: PubMed

(utilization [TIAB] OR utilisation [TIAB] OR usage [TIAB] OR uptake [TIAB] OR use* [TIAB] OR medication use [TIAB] OR drug use [TIAB] OR medicine use [TIAB] OR coverage [TIAB] OR access* [TIAB] OR health care [TIAB] OR healthcare [TIAB] OR health service* [TIAB] OR medical care [TIAB])

AND (migrat* [TIAB] OR migrant* [TIAB] OR immigrant* [TIAB] OR ethnicity [TIAB] OR race [TIAB] OR citizenship [TIAB] OR foreigner* [TIAB] OR foreign born [TIAB] OR foreign population [TIAB] OR nationality [TIAB] OR immigration [TIAB] OR asylum seeker* [TIAB] OR refugee* [TIAB] OR country of origin [TIAB])

AND (german* [TIAB] OR german [language])
