# Supplementary material for: Inequalities in health care utilization among migrants and non-migrants in Germany: a systematic review
Source: Int J Equity Health. 2018 Nov 1;17:160. doi: 10.1186/s12939-018-0876-z (PMC6211605; doi:10.1186/s12939-018-0876-z)
Supplement: Supplementary file 2 — Overview of the characteristics of included studies (utilization of outpatient care (physicians), inpatient care, emergency care and rehabilitation). (DOCX 31 kb) [file 12939_2018_876_MOESM2_ESM.docx]

**Additional file 2:** Utilization of outpatient care (physicians), inpatient care, emergency care and rehabilitation

| **Author, year [reference]** | **Sample  characteristics^a^** | **Indicator of  migrant background** | **Indicator of  health care utilization** | **Adjustments**  **(full model)** | **Statistics** | **Findings^b^** |
| --- | --- | --- | --- | --- | --- | --- |
| *Outpatient care (unspecific)* | | | | | | |
| Aparicio et al., 2005 [17] | n=4261 (natives and Eastern European immigrants of German origin), region of Augsburg, 2000 | Country of birth | Physician consultation within the past 12 months | Age, gender, partnership, education, occupation | OR^c^ | No significant difference between PMB^d^ and NMP^e^: 0.99 (0.68–1.45) |
| Bächle et al. 2010 [18] | n=21 502 (children and adolescents with type 1 diabetes), national, 2000-2008 | Nationality | Number of outpatient visits | Age, gender, year, diabetes duration, pre/post introduction of DRGs | RR^f^ | Higher utilization among PMB:  1.07 (p<0.05) |
| Fassmer et al. 2016 [19] | n=3679 (adolescents), national, 2003-2006 | Country of birth (own and parental), nationality (parental) | Number of outpatient visits within the last 12 months (cut-off: mean) | Age, smoking status, SES^g^, comorbidity | OR | Higher utilization among PMB:  1.42 (1.05–1.66) |
| Hirschfeld et al. 2015 [20] | n=2149 (children), national, 2003-2006 | Country of birth (own and parental), nationality (parental) | Physician consultation in young children with recurrent pain | Pain-related disability, -intensity, -frequency, SES, health-related quality of life, mental health problems | OR | Higher utilization among PMB:  1.64 (1.12–2.41) |
| Sundmacher et al. 2013 [21] | n=1095 (chronically ill patients), national, 2010 | Nationality | Number of consultations within the last 12 months | Age, gender, health status, hospitalization (past 12 months), education, employment status, type of health insurance, GP consultation, driving time to the nearest doctor, number of doctors per 100 000 population, spill-over effect for neighbouring regions | IRR^h^ | No significant difference between PMB and NMP: 0.92 (0.15) |
| *Outpatient care (general practitioner (GP)/paediatrician)* | | | | | | |
| Bermejo et al. 2012 [22] | n=151 Migrants and n=151 Germans (with a positive 12-month prevalence of mental disorders), national, 1997-1999 | Country of birth, nationality | Consultation of GP within the past 12 months (incl. frequency) | Matching of the two subsamples regarding gender, region, main wage earner, SES, marital status, education, age | %, M^i^ | No significant difference between PMB and NMP. Consultation PMB: 75%; NMP: 73.3%. Frequency: 5.2 (6.0)/5.0 (5.0) (p=0.583) |

| Glaesmer et al. 2011 [23] | n=2510, national, 2007 | Country of birth (own and parental) | Probability and frequency of GP utilization | Age, gender | OR, F^j^ | No significant difference between PMB and NMP regarding probability (no data shown). 1^st^ generation more frequently use general practitioners (GPs) than the native-born Germans and the 2^nd^ generation immigrants (F=3.57, p<0.05) |
| --- | --- | --- | --- | --- | --- | --- |
| Huber et al. 2012 [24] | n=17 171 (children and adolescents), national, 2003-2006 | Country of birth (own and parental), nationality (parental) | Utilization of primary care physician/GP or paediatrician within the past 12 months | Age, gender, type of health insurance, SES | OR | Lower utilization among PMB:  0.80 (0.68–0.93) |
| Kamtsiuris et al. 2007 [25] | n=approx. 17 418 (children and adolescents), national, 2003-2006 | Country of birth (own and parental), nationality (parental) | Utilization of GP and paediatrician within the past 12 months | - | % | Paediatrician: Higher utilization among PMB (63.3 vs 58.9; p<0.001). GP: Higher utilization among NMP (35.6 vs 25.6, p<0.001) |
| Zeeb et al. 2004 [26] | n=565, city of Bielefeld, 2002 | Country of birth (own and parental) | Utilization of GP within the past 6 months | - | % | Women: Higher utilization among NMP (79 vs 67.5, p=0.01).  Men: No significant difference (PMB: 52.6, NMP: 67.8, p=0.13) |
| *Outpatient care (specialist)* | | | | | | |
| Aarabi et al. 2017 [27] | n=112 (elderly ≥60 years), Hamburg metropolitan area, 2012-2014 | Country of birth | Dentist visit at least once within the last year | - | % | Higher utilization among NMP (88.2 vs 68.9, p=0.014) |
| Bermejo et al. 2012 [22] | n=151 Migrants and n=151 Germans (with a positive 12-month prevalence of mental disorders), national, 1997-1999 | Country of birth, nationality | Consultation of specialists (specialist in internal medicine, neurologist, gynaecologist, orthopaedist) within the past 12 months (incl. frequency) | Matching of the two subsamples regarding gender, region, main wage earner, SES, marital status, education, age | %, M | No significant difference between PMB and NMP in consultations and frequency regarding all types of specialists (p=0.37 to 0.98) |

| Brenne et al. 2015 [28] | n=6621-7100 (women), city of Berlin, 2011-2012 | Country of birth (own and parental) | Utilization of prenatal care | Age, parity, education, income, smoking, residence permit status, prematurity | RR, % | No significant difference concerning high utilization (≥10). 1^st^ generation*: RR 0.97 (0.92–1.02). 2^nd^ and third generation: RR 1.02 (0.97–1.06). Binational: 1.00 (0.93–1.06). Low utilization (n<5; n=644) more prevalent among migrants. 1^st^ generation: 11.8%, 2^nd^ and 3^rd^: 8.6%, NMP 7.1%). Late first utilization (≥19 week of gestation) more prevalent among 1^st^ generation migrants (7.9%) than 2^nd^ or 3^rd^ (3.5%) natives (2.2%) or binational (1.6%) |
| --- | --- | --- | --- | --- | --- | --- |
| David et al. 2014 [29] | n = 3703 (German and Turkish women), city of Berlin, 2011-2012 | Country of birth (own and parental), nationality, mother tongue | Frequency of prenatal care | - | n (M) | No significant difference between PMB and NMP: NMP 1.4 antenatal check-ups, binational women 11.3, 1^st^ generation 10.6, 2^nd^/3^rd^ generation 11.2. No significant differences in regression analyses (data not shown) |
| David et al. 2006 [30] | n=152 193 (women), city of Berlin, 1993–1999 | Nationality | Low utilization of antenatal check-ups (<11). | - | % | Lower utilization among PMB in all subgroups (lower/middle class and primipara/multipara) (p<0.01) |
| Glaesmer et al. 2011 [23] | n=2510, national, 2007 | Country of birth (own and parental) | Probability and frequency of specialist utilization | Age, gender | OR, F | Probability: Lower utilization among 1^st^ generation migrants: 0.58 (0.40–0.83). No significant difference regarding second generation: 0.75 (0.45–1.23). Frequency: No significant differences |
| Gruber & Kiesel 2010 [31] | n=2260 (elderly ≥50), national, 2004 | Country of birth | Probability and number of specialist visits within the last 12 months | Type of health insurance, size of residency area, income, education, employment status, health status, retirement | Logit, ZTNB^k^ | No significant difference in case of probability (0.115), but lower frequency among PMB (ZTNB: −0.382, p<0.01) |

| Kamtsiuris et al. 2007 [25] | n=ca. 17 418 (children and adolescents), national, 2003-2006 | Country of birth (own and parental), nationality (parental) | Utilization of specialists (specialist in internal medicine, ophthalmologist, ear, ENT (ear, nose, throat) specialist, surgeon, dermatologist, gynaecologist) within the past 12 months | - | % | Higher utilization among NMP: ophthalmologist (p<0.001), surgeon (p<0.001), gynaecologist (p=0.036). No significant differences: internal medicine (p=0.586), ENT specialist (p=0.303), dermatologist (p=0.333) |
| --- | --- | --- | --- | --- | --- | --- |
| Kavuk et al. 2006 [32] | n=471 (German and Turkish employees of a large company in Germany), 2001-2002 | Country of birth, nationality | Consultation of headache specialists | Age, gender, education, occupation, overuse of acute headache medication. | % | No utilization by 1^st^ generation migrants (2^nd^ generation: 2.8; natives: 8.8) (p<0.001) |
| Koller et al. 2009 [33] | n= 75 955 (women from Germany, Eastern Europe and Mediterranean countries), federal state of Bavaria, 2004 | Country of origin | Low utilization of prenatal check-ups (<10) | Age, partnership, occupational status, regional density of social welfare recipients, size of community | OR | Lower participation of prenatal check-ups among mothers from Eastern Europe (1.60, 1.41-1.82) and from the Mediterranean countries (1.53, 1.32-1.77) |
| Reime et al. 2009 [34] | n=182 444 (women), federal state of Lower Saxony, 1990, 1995 and  1999 | Nationality (stratified into areas of origin) | Low utilization of prenatal care (initiation of prenatal care after 12 weeks of gestation and utilization of less than half of the visits recommended until birth) | - | % | Low utilization is more prevalent among PMB: 5.9-18.6 (variations regarding area of origin; natives: 2.5) |
| Simoes et al. 2009 [35] | n=88 874 (women), federal state of Baden-Wuertemberg, 2001 | Nationality | Low utilisation of prenatal care (≤1 and 2-5) | - | RR | Lower utilisation among PMB (≤1 check-up, married: 1.77, 1.38-2.28; unmarried: 5.12, 3.11–8.46 and 2-5 check-ups, married: 2.0, 1.84 –2.17; unmarried: 3.64, 2.96-4.51) |
| Spallek et al. 2014 [36] | n=144 600 (German and mostly Turkish women) (1993-1997) and n=147 559 women (2003–2007), city of Berlin | Country of birth (own and parental) | Low number of consultation (<8) and late first consultation of prenatal care (>12 gestational week) | - | % | Significant lower number of consultation and later first consultation among PMB in both periods in nearly all subgroups (primi- and multipara, with and without partner) (p=0.022 to p<0.001) |
| Zeeb et al. 2004 [26] | n=565, city of Bielefeld, 2002 | Country of birth (own and parental) | Utilization of prenatal care, dental care and psychiatric care within the past 6 months | - | % | Lower utilization of dental care only among migrant women (p=0.02) and of prenatal care (p<0.01). No differences in case of psychiatric care |
| *Inpatient care* | | | | | | |
| Aparicio et al., 2005 [17] | n=4261 (natives and Eastern European immigrants of German origin), region of Augsburg, 2000 | Country of birth | Hospitalizations within the past 12 months | Age, gender, partnership, education, occupation | OR | No significant difference between PMB and NMP: 1.17 (0.82-1.67) |
| Bächle et al. 2010 [18] | n=21 502 (children and adolescents with type 1 diabetes), national, 2000-2008 | Nationality | Hospitalizations | Age, gender, year, diabetes duration, pre/post introduction of DRGs | RR | Higher utilization among PMB:  1.21 (p<0.05) |
| David et al. 2015 [37] | n=6875 city of Berlin, 2011-2012 | Country of birth (own and parental) | Elective caesarean section | Age, education, high-risk pregnancy, parity, language proficiency, birth weight | OR | No significant difference between PMB and NMP (ref., 1.00): 1^st^ generation: 0.88 (0.74-1.05), 2^nd^ and 3^rd^ generation: 0.80 (0.63-1.02) |
| David et al. 2014 [29] | n = 4595 (German and Turkish women), city of Berlin, 2011-2012 | Country of birth (own and parental), nationality, mother tongue | Elective caesarean section | - | % | Higher rates among NMP: 1^st^ generation: 10.5, 2^nd^ generation: 12.2, binational 13.6: non-migrants: 15.4 |
| David et al. 2006 [30] | n=152 193, city of Berlin, 1993–1999 | Nationality | Elective caesarean section | - | % | Higher rates among NMP in all subgroups (lower/middle class and primipara/multipara) (p<0.01) |
| Geyer et al. 2002 [38] | n=48 412 (children and adolescents ≤15 years), Mettmann (area west of the city of Duesseldorf), 1987-1995 | Nationality | Hospitalizations | Gender, occupational position of the main wage earner | RR | Lower hospitalisation among PMB for infections of the upper respiratory tract (0.84; 0.77-0.92) and respiratory organs (0.73; 0.64-0.83), chronic obstructive airways diseases (0.78; 0.63- 0.95) and neuroses, anxiety, depressive disorders (0.43; 0.30 to 0.61). Higher hospitalisation among PMB for pneumonia/flu (1.48; 1.21-1.80) |
| Glaesmer et al. 2011 [23] | n=2510, national, 2007 | Country of birth (own and parental) | Probability and frequency of hospitalization | Age, gender | OR, F | Probability: No significant difference between PMB and NMP. Frequency: 1^st^ generation had significantly extended hospitalization than the native-born Germans and the 2^nd^ generation immigrants (F=6.97, p<0.05) |
| Icks et al. 2007 [39] | n=1277 (diabetic children and adolescents <20 years), national, 2002-2005 | Nationality, parental country of birth, ability to speak German | Hospitalizations | Age, gender | RR | Higher risk of hospitalization among PMB: 1.17 (1.05-1.30) |
| Kamtsiuris et al. 2007 [25] | n=ca. 17 418 (children and adolescents), national, 2003-2006 | Country of birth (own and parental), nationality (parental) | Tonsillectomy, adenoidectomy, excision of the prepuce | - | % | Surgery (general): Lower utilization among PMB (33.8 vs 37.7). Adenoidectomy: Lower utilization among PMB (10.6 vs 15.9). Tonsillectomy, hernia, appendectomy: No difference. Excision of the prepuce: Lower utilization among NMP (15.3 vs 9.9) |
| Zeeb et al. 2004 [26] | n=565, city of Bielefeld, 2002 | Country of birth (own and parental) | Hospitalizations within the past 6 months | - | % | No significant differences (p=0.31 to 0.67) |
| *Emergency care* | | | | | | |
| Kietzmann et al. 2017 [40] | n=1683, national, 2014 | Country of birth (own and parental), nationality | Utilization of emergency services | Age, gender, marital status, number of children under 18, education, occupation, health status, knowledge about emergency call procedure | RR | Higher utilization among PMB: 1.49 (1.22–1.82). Lower utilization rate among 1^st^ generation migrants than 2^nd^ generation: 0.79 (0.63–1.00) |
| Wenner et al. 2016 [41] | n=ca. 17 245 (children and adolescents), national, 2003-2006 | Country of birth (own and parental), nationality (parental) | Utilization of emergency services | Age, gender, residence status, education, size of dwelling | OR | No significant difference between BMP and NMP (ref., 1.00): 0.91 (0.65-1.28) and no significant difference between 1^st^ and 2^nd^ generation (ref., 1.00): 1.06 (0.64-1.78) |
| Zeeb et al. 2004 [26] | n=565, city of Bielefeld, 2002 | Country of birth (own and parental) | Utilization of emergency services | - | % | No significant differences (p=0.17 to 1.00) |
| *Rehabilitation (out- and inpatient)* | | | | | | |
| Brzoska et al. 2010 [42] | n=19 521, national, 2002–2004 | Nationality | Utilization of rehabilitation | Age, gender, education, occupational status, income, 12 months, health status | OR | Lower utilization among PMB:  0.66 (0.49-0.90) |
| Ritter et al. 2017 [43] | n=9232 (patients with hip or knee arthroplasty), national and federal state of Baden-Wuertemberg, 2005-2010 | Nationality | Utilization of rehabilitation (hip and knee arthroplasty) | Age, gender, type of health insurance, indication, length of inability to work, comorbidity, treatment characteristics (number of consultations, cost of medication and stay, therapeutic treatments) | OR | Lower utilization among PMB:  0.54 (0.45-0.65) (hip); 0.51 (0.44-0.60) (knee) |
| Zeeb et al. 2004 [26] | n=565, city of Bielefeld, 2002 | Country of birth (own and parental) | Utilization of rehabilitation | - | % | No difference between PMB and NMP (p=0.74-1.00) (overall very low usage within the total sample) |
| Zollmann et al. 2016 [44] | n=128 165 (patients in psychosomatic rehabilitation), 2012 | Nationality | Utilization of rehabilitation | Age | n | Overall, there is no consistent pattern. Even more uptake among Turkish migrants (49.1; age-adjusted uptake ratios per 10 000). Lower rates among Germans (40.1) and people with other nationality (21.6) |

^a^Net sample (adults if not stated otherwise), area, year of data collection, ^b^Full model if not stated otherwise (significance based on 5% level), ^c^Odds ratio (confidence interval in parentheses), ^d^People with migrant background, ^e^Non-migrant population, ^f^Relative risk (confidence interval in parentheses), ^g^Socioeconomic status, ^h^Incident rate ratio (standard error in parentheses), ^i^Mean (standard deviation in parentheses), ^j^Analysis of variance, ^k^Zero truncated negative binomial regression
