# Supplementary material for: Inequalities in health care utilization among migrants and non-migrants in Germany: a systematic review
Source: Int J Equity Health. 2018 Nov 1;17:160. doi: 10.1186/s12939-018-0876-z (PMC6211605; doi:10.1186/s12939-018-0876-z)
Supplement: Supplementary file 3 — Overview of the characteristics of included studies (utilization of therapists and counselling services, complementary and alternative medicine (CAM) and medication use). (DOCX 20 kb) [file 12939_2018_876_MOESM3_ESM.docx]

**Additional file 3:** Utilization of therapists and counselling services, complementary and alternative medicine (CAM) and medication use

| **Author, year [reference]** | **Sample  characteristics^a^** | **Measurement of  migrant background** | **Indicator of  health care utilization** | **Adjustments**  **(full model)** | **Statistics** | **Findings^b^** |
| --- | --- | --- | --- | --- | --- | --- |
| *Utilization of therapists and counselling services* | | | | | | |
| Bermejo et al. 2012 [22] | n=151 Migrants and n=151 Germans (with a positive 12-month prevalence of mental disorders), national, 1997-1999 | Country of birth, nationality | Utilization of psychotherapy in the past 12 months | Matching of the two subsamples regarding gender, region, main wage earner, SES^c^, marital status, education, age | %, M^d^ | No significant difference between PMB^e^ and NMP^f^. Consultation PMB: 8.6%; NMP: 7.5%. Frequency: 15.8 (15.8) and 22.2 (18.9) (p=0.583) |
| Brenne et al. 2015 [28] | n=7100 (women), city of Berlin, 2011-2012 | Country of birth (own and parental) | Utilization of counselling services and non-medical support during pregnancy | Age, parity, education, income, smoking, residence permit status, prematurity | % | Higher utilization among NMP for diverse support services but not in case of pregnancy counselling |
| Häfner & Schmidt- Lachenmann 2008 [48] | n=481, city of Stuttgart, 1999 | Nationality | Use of psychosocial care in the past 12 months | - | % | Higher utilization among NMP:  6.5 vs 2.6 |
| Rommel & Kroll 2017 [45] | n=7987, national, 2008–2011 | Country of birth (own and parental) | Utilization of physical therapy in the past 12 months | Age, sex, education, smoking, social support, steady partnership, income, insurance status, hospitalization, medical rehabilitation, physician contacts, therapist density | OR^g^ | Lower utilization among PMB of the 1^st^ generation: 0.67 (0.51-0.89), 2^nd^ generation: 0.80 (0.60-1.08) |
| Weber et al. 2016 [47] | n= 9716 (children 3 to 13 years), national, 2003-2006 | Country of birth (own and parental), nationality (parental) | Use of occupational therapy in the past 12 months | Gender, SES, diverse health limitations, mental disorders (stratified for age) | OR | Significantly lower utilization among PMB in the age group 3-6 years (0.2; 0.1-1.0). No significant differences among age groups 7-10 years (0.5, 0.2-1.4) and 11-13 years (0.6, 0.1-3.9) |
| Weber et al. 2016 [46] | n=15 016 (children and adolescents 0 to 17 years), national, 2003-2006 | Country of birth (own and parental), nationality (parental) | Use of physical therapy in the past 12 months | Gender, SES, diverse health limitations, mental disorders (stratified for age) | OR | Significantly lower utilization among PMB in the age group 0-2 years (0.5, 0.2-1.0). Among all other age groups, just a trend but no significant differences |
| Zeeb et al. 2004 [26] | n=565, city of Bielefeld, 2002 | Country of birth (own and parental) | Utilization of psychosocial counselling in the past 6 months | - | % | Women: Higher utilization among NMP (7 vs 0, p=0.02). Men: No difference (0 vs 1.3, p=1.00) |

| Zeissig et al. 2015 [49] | n=6143 (cancer survivors), six German counties, 1994-2004 | Nationality, mother tongue, country of birth (own and parental) | Use of psycho-oncologists (hospital or in a community-based service) and patient support groups | Age, gender, income, education,  size of community, stage of disease. | OR | No significant difference between PMB and NMP (ref., 1.00). Hospital services: 1.0 (0.7-1.4), community services: 0.7 (0.4-1.3), patient support groups 0.8 (0.5-1.3) |
| --- | --- | --- | --- | --- | --- | --- |
| *Medication use (incl. complementary and alternative medicine (CAM))* | | | | | | |
| Du et al. 2009 [53] | n=17 450 (children and adolescents 0 to 17 years), national, 2003-2006 | Country of birth (own and parental), nationality (parental) | Use of homoeopathic medicines in the last 7 days | Age, gender, region, urbanicity, health status, maternal and paternal education, SES, breast feeding, smoking and drinking during pregnancy | OR | Higher utilization among NMP: 3.66 (2.21–6.06) |
| Du et al. 2009 [50] | n=17 450 (children and adolescents 0 to 17 years), national, 2003-2006 | Country of birth (own and parental), nationality (parental) | Use of self-medication in the last 7 days | Age, gender, region, urbanicity, health status, household income, parental education | OR | Higher utilization among NMP. Overall: 1.55 (1.33-1.80), over the counter drugs: 1.88 (1.56-2.27), other sources: 1.16 (0.96-1.40) |
| Du et al. 2014 [54] | n=17 450 (children and adolescents 0 to 17 years), national, 2003-2006 | Country of birth (own and parental), nationality (parental) | Use of herbal medicinal products in the last 7 days | Age, gender, region, urbanicity, BMI, health status, children with special health care needs-screener | OR | Higher utilization among NMP: 1.65 (1.28-2.11) |
| Eckel et al. 2014 [51] | n=17 450 (children and adolescents 0 to 17 years), national, 2003-2006 | Country of birth (own and parental), nationality (parental) | Self-medicated and prescribed drug use (medicines to treat common colds and upper respiratory tract infections) in the last 7 days | Age, gender, region, SES | OR | Self-medication: Higher utilization among NMP (ref. PMB, 1.00): 1.64 (1.28-2.11).  Prescribed medicine: Higher utilization among PMB (ref. NMP, 1.00): 1.60 (1.21-2.11). Antibiotic use: Higher utilization among PMB (ref. NMP, 1.00): 2.37 (1.51-3.73) |
| Huber et al. 2012 [24] | n=17 171 (children and adolescents 0 to 17 years), national, 2003-2006 | Country of birth (own and parental), nationality (parental) | Use of alternative practitioner in the past 12 months | Age, gender, insurance status, SES | OR | Lower utilization among PMB: 0.58 (0.40-0.83) |
| Kalder et al. 2011 [55] | n=205 (women), region of Giessen, 2007-2008 | Nationality | Use of complementary and alternative medicine (CAM) during pregnancy and delivery | not shown | F^h^ | Higher utilization among NMP:  F= 13.8 (p<0.001) |
| Kavuk et al. 2006 [32] | n=471 (German and Turkish employees of a large company), 2001-2002 | Country of birth, nationality | Use of headache preventive medication and of non-medical treatment options (including consultation with an Islamic priest) within one year (chronic headache) | - | % | No utilization of preventive medication among PMB (natives: 6.6; p=0.014). Higher utilization of non-medical treatment options among Turkish migrants of the 1st generation: 28.4 vs 4.5 (natives) (p=0.001) |
| Knopf 2007 [52] | n=17 450 (children and adolescents 0 to 17 years), national, 2003-2006 | Country of birth (own and parental), nationality (parental) | Medicine use in the last 7 days | - | % | Children from migrant families stated a less frequent use of medication (41.5 vs 52.8) |
| Knopf et al. 2008 [58] | n=17 641 (children and adolescents 0 to 17 years), national, 2003-2006 | Country of birth (own and parental), nationality (parental) | Use of tablet fluoridation (dental care) | Age, gender, size of community, region, SES | OR | Higher utilization among NMP:  1.9 (1.5-2.4) |
| Knopf et al. 2012 [57] | N=4834 (children and adolescents 6 to 17 years), national, 2003-2006 | Country of birth (own and parental), nationality (parental) | Attention-deficit hyperactivity disorder (ADHD) medication use in the last 7 days | Age, gender, size of community, region, SES, urbanity, health status, tobacco smoking and alcohol consumption during pregnancy, last visit to a doctor | OR | Higher utilization among NMP:  3.06 (1.34-6.99). Children with ADHD diagnosis (n=518): 1.22 (0.39 -3.81) |
| Knopf et al. 2013 [59] | n=7808 (children and adolescents 0 to 17 years), national, 2003-2006 | Country of birth (own and parental), nationality (parental) | Off-label medicine use in the last 7 days | Age, gender, region, urbanity, SES, health status | OR | No significant difference between PMB and NMP (ref., 1.00): 0.99 (0.82-1.21) (children with off-label vs. excl. in-label medicine use) |
| Mani et al. 2015 [56] | n=172 (patients before and after organ removal due to urologic cancer), city of Frankfurt, 2007-2011 | Nationality | Use of CAM in the 2 years before and in the 2 years after surgery | - | % | Higher utilization among NMP (n=56 CAM users): 93 vs. 7 (p=0.036) |
| Zeeb et al. 2004 [26] | n=565, city of Bielefeld, 2002 | Country of birth (own and parental) | Utilization of CAM and medication use in the past 6 months | - | % | Women: Higher utilization of CAM (p<0.01) and higher medication use (p<0.01) among women of NMP |

^a^Net sample (adults if not stated otherwise), area, year of data collection, ^b^Full model if not stated otherwise (significance based on 5% level), ^c^Socioeconomic statu, ^d^Mean (standard deviation in parentheses), , ^e^People with migrant background, ^f^Non-migrant population, ^g^Odds ratio (confidence interval in parentheses), ^h^Analyses of variance
