# Supplementary material for: Inequalities in health care utilization among migrants and non-migrants in Germany: a systematic review
Source: Int J Equity Health. 2018 Nov 1;17:160. doi: 10.1186/s12939-018-0876-z (PMC6211605; doi:10.1186/s12939-018-0876-z)
Supplement: Supplementary file 4 — Overview of the characteristics of included studies (utilization of disease prevention). (DOCX 28 kb) [file 12939_2018_876_MOESM4_ESM.docx]

**Additional file 4:** Utilization of disease prevention

| **Author, year [reference]** | **Sample  characteristics^a^** | **Operationalization of  migrant background** | **Indicator of  health care utilization** | **Adjustments**  **(full model)** | **Statistics** | **Findings^b^** |
| --- | --- | --- | --- | --- | --- | --- |
| *Early detection (cancer)* | | | | | | |
| Aparicio et al., 2005 [17] | n=4261 (natives and Eastern European immigrants of German origin), region of Augsburg, 2000 | Country of birth | Non-participation in early detection (any cancer) in the past 12 months | Age, gender, partnership, education, occupation | OR^c^ | Higher non-participation among PMB^d^: 2.46 (1.77-3.41) |
| Berens et al. 2014 [60] | n=208 500 (German and Turkish women), five screening units from different regions, 2010-2011 | Identification by using a name-based algorithm | Participation in breast cancer screening | Age, screening unit | OR | Higher chance to participate among PMB: 1.17 (1.14-1.21) |
| Brand et al. 2015 [61] | n=22 048, national, 2009-2010 | Country of birth (own and parental), nationality | Participation in cancer screening programs | - | % | Lower participation among PMB: (40 vs 51). No change after stratification for SES^e^. |
| Brzoska &  Abdul-Rida 2016 [62] | n=11 709 (women aged ≥20 years), national, 2008-2009 | Country of birth (own and parental), nationality | Participation in cancer screening programs | Age, education, occupational status, income, relationship status, health status, comorbidity, prior cancer diagnosis, smoking status | OR | Lower participation among PMB with a two-sided migration background: 0.55 (0.47-0.64) (one-sided migration background: 0.98; 0.76-1.26) |
| Lemke et al. 2015 [63] | Numbers of participants (women between 50 and 69 years) by statistical district were aggregated over the three periods, city of Dortmund, 2007-2012 | Nationality | Participation in breast cancer screening | - | OR | No significant difference between PMB and NMP^f^: 0.95 (0.89-1.02) (crude model only) |
| Rommel et al. 2015 [64] | n=7987, national, 2008-2011 | Country of birth (own and parental) | No regular participation in cancer screening programs (based on national recommendations) | Age, SES | OR | Less regular participation among 1^st^ generation female migrants: 1.45 (1.10–1.92) (2^nd^ generation: 1.29; 0.91-1.83). No significant associations among males (1^st^ generation: 1.28; 0.84-1.95 and 2^nd^ generation: 0.93; 0.60-1.43) |

| Zeeb et al. 2004 [26] | n=565, city of Bielefeld, 2002 | Country of birth (own and parental) | Participation in cancer early detection in the past 6 months | - | % | Women: Higher utilization among NMP (90 vs 61, p<0.01). Men: No difference (12.9 (NMP) vs 11.8, p=0.25) |
| --- | --- | --- | --- | --- | --- | --- |
| *Early detection (children)* | | | | | | |
| Becker & Kurz 2011 [65] | n=1028 (mothers with newborns aged 0 to 18 months), national, 2003-2008 | Country of birth (own and parental) | Participation in the preventive health care program for children (U-examinations) | Age and gender of child, year of birth (child), age of mother at birth, region, pregnancy was planned or not, coping competencies and time constraints, family structure and social support, health status of child and of mother during pregnancy | OR | Lower utilization among PMB:  0.47 (p<0.01) |
| Kamtsiuris et al. 2007 [25] | n=approx. 17 418 (children and adolescents 0 to 17 years), national, 2003-2006 | Country of birth (own and parental), nationality (parental) | Participation in the preventive health care program for children (U3 to U9) | - | % | Lower utilization among PMB for every single diagnostic test (U3 to U9) (p<0.001) |
| Koller & Mielck 2009 [66] | N=9 353 (birth cohort of 6-year old children), city of Munich, 2004 | Mother tongue of the parents | Participation in the preventive health care program for children (U1 to U9; at least one of the nine check-ups missing) | Gender and kindergarten visit (individual level), low education households and single parent households (district level) | OR | More incomplete check-ups among PMB: 2.94 (2.63-3.28) |
| Rosenkötter et al. 2012 [67] | n=52 171 (children), federal state of North Rhine-Westphalia, 2007 | Language spoken at home | Participation in the preventive health care program for children (U3 to U9; at least one of the nine check-ups missing) | - | OR | More incomplete check-ups among PMB: 3.71 (3.56–3.87) (crude effect only) |
| Stich et al. 2009 [68] | n= 1972 (children), district of Dingolfing-Landau, 2004-2005 | Nationality, country of birth (own and parental), mother tongue | Participation in the preventive health care program for children (U1 to U8) | Every significant variable from univariate regression was included in the adjusted model: Smoking status (parents), nationality, country of birth (parents), mother tongue, living space/person, satisfaction with job, satisfaction with financial situation | OR | More incomplete check-ups among PMB: Univariate analyses: Nationality: 4.74 (3.32-6.78). Parental country of birth: 2.37 (1.13-4.99) (one parent born in Germany), 6.15 (3.96-9.54) (both parents born abroad). No German mother tongue: 7.67 (5.12-11.49). Multivariate analyses (only significant): No German mother tongue: 4.36 (2.73-6.96) |
| *Vaccination* | | | | | | |
| Bödeker et al. 2014 [69] | n=1025 (pregnant women), national, 2013 | Country of birth (own and parental) | Seasonal influenza vaccine uptake and pertussis vaccination within the last 10 years | Education, place of residence, comorbidity, first pregnancy, use of alternative medicine, influenza vaccination history, annual receipt of seasonal influenza vaccination (pertussis only), knowledge and perception of the disease and vaccination effects (influenza only); analyses weighted for age and region | OR | Influenza: No significant difference between PMB and NMP. Univariate analyses: 1.33 (0.87-2.04), multivariate analyses (n=994): not significant (data not shown). Pertussis: Lower uptake among PMB: 0.52 (0.33–0.83) (multivariate) |
| Böhmer et al. 2011 [70] | n=21 262, national, 2008-2009 | Country of birth (own and parental), nationality | Tetanus vaccination status (last tetanus shot > or ≤10 y ago) among general population, influenza vaccination coverage in recommended target population (i.e., persons ≥60 years, chronically ill, healthcare workers) | Age, gender, region, size of community, type of health insurance, received vaccination advice, information about vaccination, last physician contact, socioeconomic status, health awareness, health behaviour, comorbidity, health status | OR | Lower uptake of tetanus vaccination among PMB (two-sided): 0.60 (0.53-0.67). Higher uptake of seasonal influenza vaccination among PMB (two-sided): 1.23 (1.05-1.44). No significant results for people with one-sided migrant background |
| Brand et al. 2015 [61] | n=22 048, national, 2009-2010 | Country of birth (own and parental), nationality | Uptake of seasonal influenza in 2008/2009 | - | % | Lower uptake of influenza vaccination (17 vs 25) among PMB. No change after stratification for SES |
| Koller & Mielck 2009 [66] | n=9353 (birth cohort of 6-year old children), city of Munich, 2004 | Mother tongue of the parents | Incomplete immunizations (at least one out of the nine vaccinations missing) | Gender and kindergarten visit (individual level), low education households and single parent households (district level) | OR | Less incomplete immunizations among PMB: 0.60 (0.55–0.66) |
| Mikolajczyk et al. 2008 [71] | n=1426 (pre-school children), rural region in Bavaria, 2004- 2005 | Acculturation index (child: country of birth, citizenship, native language, language spoken with friends; respondent: age by entry in the country, age of the partner by entry in the country, language spoken with the child) | Vaccination coverage of measles-mumps-rubella (MMR) (lacking) and hepatitis B (incomplete (1–2 doses) or lacking) | Gender of the child, education of the parents, bringing up the child (both parents/single parent), size of apartment, satisfaction with financial situation, number of children in the household | OR | No difference between PMB (more acculturated and less acculturated) and NMP in case of lacking MMR. Increased lacking (0.30; 0.12-0.77) and incomplete (2.74; 1.34-5.61) hepatitis B vaccination among less acculturated migrants in case of hepatitis B |

| Poethko- Müller et al. 2009 [72] | n=14 826 (children and adolescents 2 to 17 years), national, 2003-2006 | Country of birth (own and parental), nationality (parental) | Vaccination coverage against measles | Age, gender, place of residence, socioeconomic status, number of siblings, attitude to vaccination | OR | Lower uptake among PMB of the 1^st^ generation: 3.03 (2.06-4.45). No association with 2^nd^ generation migrants and no clear pattern when stratified by one- or two sided migration background |
| --- | --- | --- | --- | --- | --- | --- |
| Poethko- Müller et al. 2007 [73] | n=16 460, national, 2003-2006 | Country of birth (own and parental), nationality (parental) | Vaccination rates (tetanus, diphtheria, Hib and poliomyelitis, hepatitis B, pertussis, measles-mumps-rubella (MMR)) | - | % | Lower uptake for tetanus (primary and booster), diphtheria (primary and booster), Hib and poliomyelitis among PMB. No striking difference for hepatitis B, pertussis and MMR |
| Remschmidt et al. 2014 [75] | n=823 (women), national, 2010-2012 | Parental country of birth | HPV-vaccine uptake | Age, current smoking, education | OR | Lower uptake among PMB: 0.4 (0.2-0.8) |
| Remschmidt et al. 2014 [74] | N=1161 (women recruited via a social media site), national, 2012-2013 | Parental country of birth | HPV-vaccine uptake | Age, region, education, religion, HPV-vaccination recommended by physician, vaccinated against tetanus, level of being informed about HPV vaccines, actively sought information on HPV vaccination, convinced of HPV vaccine effectiveness, concerns of HPV vaccine severe side effects, feels adequately informed to opt for/against HPV vaccination, opinion that HPV-vaccination replaces cervical cancer screening, opinion that condoms protect against HPV | OR | No significant differences in crude (0.4; 0.7-1.2) and adjusted analyses (no data shown) |
| Rosenkötter et al. 2012 [67] | n=52 171 (children), federal state of North Rhine-Westphalia, 2007 | Language spoken at home | Incomplete immunisation uptake (at least one vaccination missing or insufficient) | Parental education, kindergarten visit, single parent household, area type, participation in early recognition examinations | OR | More incomplete immunisation uptake among NMP: 0.88 0.84–0.93 |
| Samkange-Zeeb et al. 2012 [76] | n=632 (girls aged 12 to 20 years), cities of Bremen and Bremerhaven, 2011 | Language spoken at home | HPV vaccination uptake | - | OR | No differences between PMB and NMP: 0.78 (0.43–1.41) (data shown for univariate analysis only) |
| Stumm et al. 2017 [77] | n=1515 (women 13 to 21 years), city of Fulda, 2011 | Nationality and parental country of birth | HPV vaccination uptake | Age, sex, education, religion | OR | Higher uptake among NMP:  2.2 (1.1–4.3) |
| Wenner et al. 2016 [41] | n=17 245 (children and adolescents), national, 2003–2006 | Country of birth (own and parental), nationality (parental) | Incomplete vaccination status (based on nation-al recommendations) | Age, sex, residence status, education, size of dwelling | OR | No differences between PMB and NMP: 1.12 (0.98-1.30). More incomplete vaccination among PMB of the 1^st^ generation than of the 2^nd^: 1.85 (1.38-2.48) |
| *General health check-up* | | | | | | |
| Brand et al. 2015 [61] | n=22 048, national, 2009-2010 | Country of birth (own and parental), nationality | Participation in general health check-up in the past 24 months | - | % | Lower participation among PMB: (40 vs 51). No change after stratification for SES |
| Zeeb et al. 2004 [26] | n=565, city of Bielefeld, 2002 | Country of birth (own and parental) | Participation in general health check-up in the past 6 months | - | % | Women: Higher utilization among NMP (40 vs 26, p<0.01). Men: No significant difference (36 (NMP) vs 29, p=0.26) |
| *Oral health check-up* | | | | | | |
| Aarabi et al. 2017 [27] | n=112 (elderly ≥60 years), Hamburg metropolitan area, 2012-2014 | Country of birth | Reason if check-up was the reason for the last visit | - | % | No difference if check-up was the reason for the last visit (70 vs 60.7, p=0.305) |
| Brand et al. 2015 [61] | n=22 048, national, 2009-2010 | Country of birth (own and parental), nationality | Participation in oral health check-ups in the past 12 months | - | % | Higher participation among NMP: 76.6 vs 66.2 |
| Erdsiek et al. 2017 [78] | n=21 741, national, 2009-2010 | Country of birth (own and parental), nationality | Utilization of oral health check-ups in the past 12 months | Age, gender, socioeconomic status, health insurance status, living together with a partner/spouse, region, urban residence | OR | Lower utilization among PMB: 0.71 (0.65 0.77) |
| Huber et al. 2012 [24] | n=17 171 (children and adolescents), national, 2003-2006 | Country of birth (own and parental), nationality (parental) | Low utilization (once a year or less) of oral health check-ups within the past 12 months | Age, gender, health insurance status, SES | OR | Higher probability of low utilization among PMB: 1.72 (1.53-1.94) |
| Knopf et al. 2008 [58] | n=17 641 (children and adolescents 0 to 17 years), national, 2003-2006 | Country of birth (own and parental), nationality (parental) | Low utilization of oral health check-ups (less than once a year) | Age, gender, size of community, region, SES | OR | Higher probability of low utilization among PMB: 2.2 (1.8-2.6) |
| Kühnisch et al. 1998 [79] | n=450 (8 year old children), district of Ennepe-Ruhr, 1997 | Nationality | Low utilization of oral health check-ups (less than twice a year) | - | % | Lower utilization among PMB: 37.8 vs 17.8 |
| Zeeb et al. 2004 [26] | n=565, city of Bielefeld, 2002 | Country of birth (own and parental) | Utilization of oral health check-ups in the past 6 months | - | % | Lower utilization among PMB for women (50 vs 79) and men (29 vs 58) |

^a^Net sample (adults if not stated otherwise), area, year of data collection, ^b^Full model if not stated otherwise (significance based on 5% level), ^c^Odds ratio (confidence interval in parentheses), ^d^People with migrant background, ^e^Socioeconomic status, ^f^Non-migrant population
